# Si-Doped HfO_2_-Based Ferroelectric Tunnel Junctions with a Composite Energy Barrier for Non-Volatile Memory Applications

**DOI:** 10.3390/ma15062251

**Published:** 2022-03-18

**Authors:** Yoseop Lee, Sungmun Song, Woori Ham, Seung-Eon Ahn

**Affiliations:** Department of Nano & Semiconductor Engineering, Korea Polytechnic University, Siheung 15073, Korea; ukaslo@kpu.ac.kr (Y.L.); 2020811029@kpu.ac.kr (S.S.); dnfl2475@kpu.ac.kr (W.H.)

**Keywords:** FTJ, ferroelectric, non-volatile memory

## Abstract

Ferroelectric tunnel junctions (FTJs) have attracted attention as devices for advanced memory applications owing to their high operating speed, low operating energy, and excellent scalability. In particular, hafnia ferroelectric materials are very promising because of their high remanent polarization (below 10 nm) and high compatibility with complementary metal-oxide-semiconductor (CMOS) processes. In this study, a Si-doped HfO_2_-based FTJ device with a metal-ferroelectric-insulator-semiconductor (MFIS) structure was proposed to maximize the tunneling electro-resistance (TER) effect. The potential barrier modulation effect under applied varying voltage was analyzed, and the possibility of its application as a non-volatile memory device was presented through stability assessments such as endurance and retention tests.

## 1. Introduction

With the advent of the information age, Internet of things (IoT)-based electronic communication equipment has produced huge amounts of data [1]. Moreover, the number of data centers that store huge amounts of data has increased exponentially, leading to a situation in which physical space cannot be ignored [2,3]. In addition, the operation of large-scale servers encounter power consumption issues because of the requirements for standby power and cooling for heat control [4]. To solve these problems, it is necessary to develop high-performance next-generation semiconductor devices [5,6,7]. In particular, the non-volatility, high integration, high operating speed, and low power consumption of memory semiconductors for information storage must be improved. Currently, commercialized dynamic random access memory (DRAM), static random access memory (SRAM), and NAND flash memory have made great progress through continuous development, but they still do not satisfy some requirements for next-generation memory devices [8]. As a consequence, the development of a new type of memory device is essential. Recently, various memory devices based on non-volatile characteristics, such as resistive switching memory [9,10], phase-change memory [11,12], ferroelectric memory [13,14], and ferromagnetic memory [15,16] have been introduced as next-generation memory device candidates. Among these various memory candidates, ferroelectric tunnel junction (FTJ) memory using ferroelectric polarization reversal is considered a promising candidate owing to its functional superiority and scalability [17,18,19,20,21]. An FTJ device modulates the potential barrier between the electrode and ferroelectric material through the polarization reversal of the ferroelectric thin film, forming two different electrical resistance states, which is known as tunneling electroresistance (*TER*) [22,23]. The *TER* effect, which is directly related to the performance of the memory device [24], is expressed as follows [25]:

(1) $$TER=\frac{J_{ON}}{J_{ON}-J_{OFF}}$$

where *J_ON_* is the current density in the ON state, and *J_OFF_* is the current density in the OFF state. The theoretical concept of FTJ was proposed in the form of a “polar switch” and was initially studied based on perovskite ferroelectrics [26,27,28,29]. To improve the electron tunneling phenomenon, it was necessary to continuously reduce the thickness of the ferroelectric thin film. However, the development of perovskite ferroelectric-based FTJ devices has been at a standstill for a long time because of the scale-down issue in which ferroelectricity is lost at ultra-thin thicknesses [30]. Recently, the ferroelectricity of HfO_2_ was reported in ultra-thin films (≤10 nm) [31]. Subsequently, many studies have applied HfO_2_-based ferroelectrics to achieve high-performance FTJ devices. In particular, HfO_2_ thin films have easy access to mass production owing to advantages such as their high compatibility with the complementary metal-oxide-semiconductor (CMOS) process and low crystallization temperature [18,30,32]. In addition, the FTJ device can be fabricated using a two-terminal structure such as for metal-ferroelectric-metal (MFM), which has the advantage of being applicable for designs for high integration of 4F^2^ [33,34,35,36]. Tunneling current-based operation and a fast polarization reversal speed enable low power consumption and fast driving [37,38]. In this study, a metal-ferroelectric-insulator-semiconductor (MFIS) stack with a composite barrier was investigated. An asymmetric potential barrier was induced by employing heavily doped n-type Si and SiON insulators. We demonstrated that the MFIS stack had a high TER effect through a memory operation characteristic analysis and an energy band diagram simulation. The memory characteristics were verified using resistance–voltage (R–V) hysteresis loops showing the resistive switching characteristics, and the reliability of the memory characteristics was evaluated. Finally, we presented the possibility of next-generation memory applications for MFIS FTJ devices.

## 2. Materials and Methods

To verify the improvement in the memory characteristics of the composite barrier FTJ device, MFM and MFIS structures were fabricated with a Si-doped HfO_2_ (HSO) ferroelectric film. The bottom TiN electrode of the MFM structure was deposited to a thickness of 20 nm through reactive sputtering with a DC plasma source (Advanced Energy, Campbell, CA, USA). The partial pressure ratio of O_2_ and N_2_ gas was (N_2_ 20 sccm/(O_2_ 5 sccm + N_2_ 20 sccm)) × 100 = 80%. The DC power was 100 W, and the working pressure was 2 mTorr. For the MFIS stack, SiON was grown to a thickness of 25 Å on heavily doped *n*-type Si (≈10^19^ cm^−3^) substrate. Si-doped HfO_2_ ferroelectric film was deposited with a thickness of 8 nm via atomic layer deposition (ALD) at 280 °C. A mixture of Hf[N(CH_3_)(C_2_H_5_)]_4_ (TEMAH) and Si[N(CH_3_)_2_]_4_ (4DMAS) (Hf:Si = 16:1) was used as the precursor, and ozone was used as the oxygen source. The composition ratio of the Si used as a dopant was 4.2 mol%. Subsequently, TiN was deposited using the same process as the bottom electrode, and Pt was deposited to a thickness of 50 nm using an e-beam evaporator. The top electrode was patterned using a circular pattern (radius = 100 µm) hard mask. Subsequently, a rapid thermal annealing (RTA) process was performed in a N_2_ atmosphere at 600 °C for 20 s.

## 3. Results

**Figure 1:**
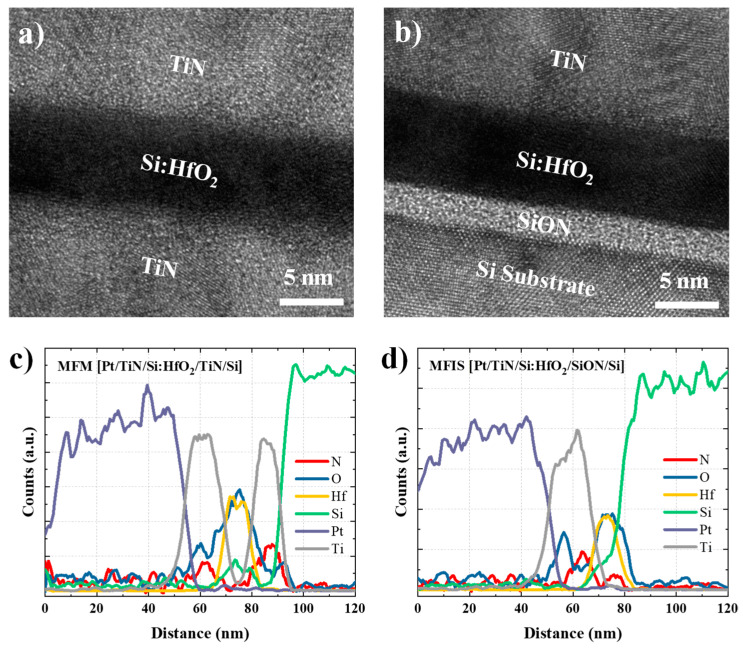
The TEM data for (**a**) MFM and (**b**) MFIS FTJ devices; elemental depth profiles of (**c**) MFM and (**d**) MFIS stacks analyzed by EDS.

Figure 1a,b show transmission electron microscopy (TEM) bright field images of the FTJ devices with MFM (TiN/HSO/TiN) and MFIS (TiN/HSO/SiON/Si) structures, respectively. It can be seen that HSO for non-volatile memory performance was deposited to a thickness of 8 nm, and SiON for asymmetric potential barrier formation was deposited to a thickness of 25 Å. Figure 1c,d show the elemental depth profiles of the TiN/HSO/TiN and TiN/HSO/SiON/Si stacks, respectively, obtained using energy dispersive spectroscopy (EDS). Because the upper stacks of the MFM and MFIS structures are identical, the depth profiles of the Pt/TiN/HSO stacks are similar. In the case of the lower stacks, the Ti and N peaks are visible under the HSO thin film in the MFM structure, whereas the Si and N peaks are increased for the MFIS structure. The TEM images and EDS element depth profiles verify that the MFM and MFIS structures are well implemented. An asymmetric electrode structure using a heavily doped n-type Si substrate and a SiON insulator was designed to maximize the TER effect by using the asymmetry of the potential barrier formed in the MFIS structure.

**Figure 2:**
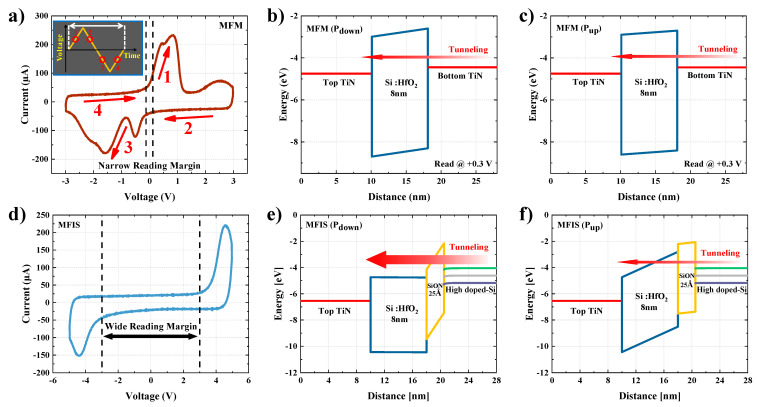
The current–voltage characteristics of (**a**) MFM and (**d**) MFIS FTJ devices measured using a triangular waveform; energy band diagrams for polarization directions of down and up in the (**b**,**c**) MFM structure and (**e**,**f**) MFIS structure.

Figure 2a,d shows the current-volatge (I-V) characteristics of the MFM and MFIS FTJ devices, respectively, measured using a triangular waveform of 2 kHz, as shown in the inset of Figure 2a. When the amplitude of the triangular waveform rises, polarization switching occurs in the opposite direction of the electric field. Through polarization switching, the amount of movement of the electric charge increases, resulting in an increase in the current. Peak points 1 and 3 of the I–V curve correspond to the switching current. Because the voltage applied to the MFM stack is applied only to the ferroelectric film, polarization switching begins at a very low voltage, and the MFM FTJ has a very narrow read voltage margin. However, in the MFIS stack, the insertion of the insulator causes a voltage division across the ferroelectric film and the insulator. In particular, because the HfO_2_ thin film used as a high-k material (ε_r_ = 25~35) has a relatively high dielectric constant compared to that of SiON (ε_r_ = 4.6), the amount of voltage division is small, even when the thin film is thicker [39,40]. Therefore, the MFIS FTJ device undergoes polarization switching at a higher voltage than the MFM FTJ device; thus, the read margin, which is an important factor in the operation of FTJ devices, is relatively large in the MFIS structure. In addition, as shown in Figure 2d, it is possible to secure a stable driving voltage range of the FTJ device with a wide read voltage margin of −2.5 V to 2.5 V that does not affect the polarization state. Figure 2b,c shows energy band diagrams when the direction of polarization in the MFM stack is down (b) and up (c). Figure 2e,f shows the energy band diagrams in the MFIS stack.

In the MFM stack, there is a barrier difference during the same read operation due to polarization reversal; however, there is no significant barrier difference due to the symmetrical electrode structure. Consequently, the TER according to the polarization direction is not expected to be large. In contrast, in the MFIS structure, the energy band change with downward and upward polarization is greater than that of the MFM stack. The potential barrier of SiON bends steeply in the case of downward polarization, and the width of the effective tunneling barrier is narrow and steep. Therefore, when the polarization is downward, the tunneling current is expected to be large. On the other hand, in upward polarization, the SiON potential barrier is not sharp, and the effective tunneling barrier is also thick; hence, a small tunneling current flows. As a result, unlike the MFM structure, the MFIS structure can maximize the TER effect because the barrier asymmetry with the polarization direction is large.

The memory properties of the FTJ device that operates based on a polarization switching mechanism are strongly associated with the changes in the polarization switching distribution and density in the ferroelectric thin film. Therefore, investigating the switching characteristics of the device through electric field cycling is important for assessing its reliability. To analyze the switching characteristics of the device, the I–V curve and first-order reversal curve (FORC) were analyzed. The FORC is an effective method for observing domain movement, and it can be used to analyze the polarization density of a ferroelectric film switched in a specific electric field. Using the saturation voltage (MFM: −3 V; MFIS: −5 V) as the base bias, triangular pulses are applied in which the reverse electric field is increased at +0.25 V per step in each subsequent sweep. The switching density (*ρ*) obtained from the difference between the current densities of *E_r,i_* and *E_r,i−_*_1_ is expressed as follows [41,42]:

(2) $$\rho^{-}\left(E_{r},E\right)\approx \frac{1}{2E}\times \frac{J_{FORC}^{-}\left(E_{r,i},E\right)-J_{FORC}^{-}\left(E_{r,i-1},E\right)}{E_{r,i}-E_{r,i-1}}$$

where *E* is the electric field, *E_r_* is the reverse electric field, *i* is the number of reversed electric field sweeps starting from the saturation voltage, and *J_FORC_* is the current density. The switching density extracted using Equation (2) is expressed as a Preisach model, and the distribution of the coercive field (*E_c_*) and internal bias field (*E_bias_*) can be observed in detail.

(3) $$E_{c}=\frac{E-E_{r}}{2},\;E_{bias}=\frac{E+E_{r}}{2}\;$$

**Figure 3:**
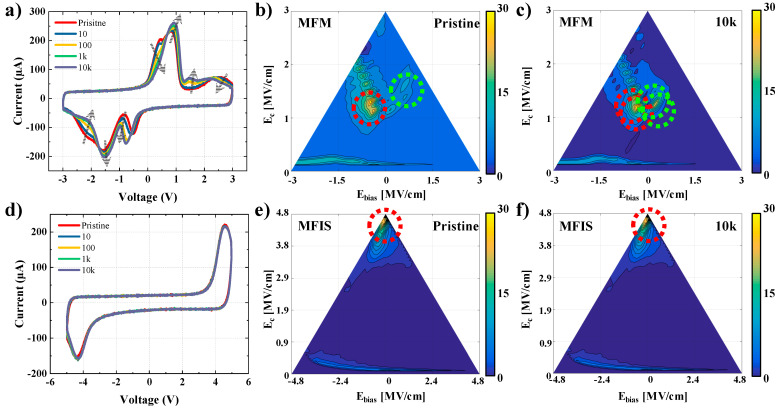
A comparison of the stability of the MFM and MFIS FTJ devices under electrical switching cycling: current–voltage curves of (**a**) MFM and (**d**) MFIS FTJ devices with an increase in the number of cycles; switching density pseudo-color plots (**b**), (**e**) in the pristine state, and (**c**), (**f**) after 10 k switching cycles. The scale bar to the right of the graph represents the switching density, *ρ*.

Figure 3a shows the I–V characteristics of the MFM FTJ device with electric field cycling. As the electric stress increases, the wake-up, switching peak shift, and merging effects occur continuously [43,44]. This can be observed in detail in the FORC diagram. Figure 3b shows a switching density pseudo-color plot of the MFM stack in the pristine state. The switching density peaks are divided into two groups (red and green circles) at *E_bias_* = −0.65 and 0.75 MV/cm, respectively. Both groups are shifted towards *E_bias_* = 0 MV/cm, which is different from the pristine state after 10^4^ electric field cycles (Figure 3c). Figure 3d shows the I-V characteristics of the MFIS FTJ device with electric field cycling. Owing to the presence of the insulator, switching occurs later, and a switching peak is observed at 4.5 V. However, there is a slight variation in the switching characteristics due to electric field cycling. Figure 3e shows a switching density pseudo-color plot of the MFIS stack in the pristine state. One switching density peak is observed near *E_bias_* = −0.2 MV/cm. Unlike the MFM stack, the switching density peak does not shift in the MFIS stack, even after 10^4^ electric field cycles (Figure 3f). These results indicate that the MFIS stack has stable switching characteristics in terms of reliability, and thus the FTJ device with a composite energy barrier has greater application potential as a memory device than the MFM FTJ device with a symmetric energy barrier. Therefore, the memory characteristics analysis was performed using the MFIS FTJ device.

**Figure 4:**
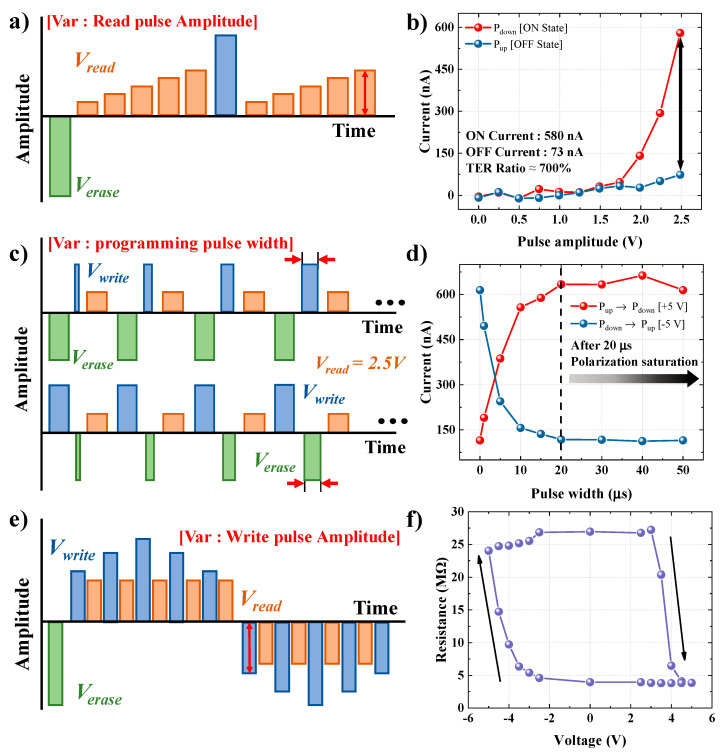
The pulse driving conditions analysis: (**a**) pulse scheme used to determine the read driving condition; (**b**) tunneling current depending on read voltage pulses with varying amplitudes and a pulse width of 100 μs; (**c**) pulse scheme used to determine the write driving condition; (**d**) tunneling current depending on the write voltage pulse; (**e**) pulse scheme used to obtain the resistance–voltage (R–V) hysteresis data; (**f**) R–V hysteresis loop.

In Figure 4, the analysis of the pulse driving conditions applicable to read and write driving for the memory operation of the FTJ device is performed. Figure 4a shows the pulse scheme used to determine the read operation. Before the read pulse, the polarization is aligned upward and downward to investigate the difference in the tunneling current. An amplitude of ±5 V and a pulse of 50 µs were used to ensure sufficient alignment of the polarization in one direction. Then, the tunneling current was measured by applying pulses from 0 V to 2.5 V at 0.5 V steps. In addition, to observe the dependence of the tunneling current on the change in amplitude, the pulse width was fixed at 100 μs. Figure 4b shows the read current as a function of the applied voltage. Below 1.75 V, there is no significant current difference between the upward and downward polarizations. However, the current difference begins to occur from 2 V, and the largest TER ratio occurs at 2.5 V. The MFIS FTJ device shows tunneling currents of 580 nA in the ON state and 73 nA in the OFF state, resulting in a TER value of 700%. This tunneling current difference is similar to that predicted by analyzing the energy band diagram shown in Figure 2. Figure 4c shows the pulse scheme used to optimize the pulse width for the writing (top) and erasing (bottom) operation. The pulse amplitude was fixed at 5 V (writing) and −5 V (erasing), and the tunneling current was measured using a read voltage pulse after each programming (write or erase) with pulses 1–50 µs in pulse width. In the Figure 4d, the red data is the measured tunneling current along the width of the write programming pulse (Figure 4c top), and the blue data is the measured tunneling current while adjusting the width of the erase programming pulse (Figure 4c bottom). Regardless of the polarization direction, the tunneling current initially increases as the pulse width increases and then saturates at 20 µs (Figure 4d). Thus, we can determine that the FTJ device completes the barrier modulation at a pulse width of 20 µs. The use of a write pulse of ±2.5–5 V, 20 µs and a read pulse of 2.5 V, 100 µs was validated by the experimental results as the driving conditions for obtaining the optimal TER effect. A resistance–voltage (R-V) hysteresis loop was investigated using the optimized pulse driving conditions. Figure 4e shows the pulse scheme used to obtain the R–V hysteresis data. The tunneling current, measured as the write pulse, was increased from 0 V → 2.5 V → 5 V and decreased from 5 V → 2.5 V → 0 V → −2.5 V → −5 V. When the negative saturation amplitude was reached, it was increased again to 0 V. The resistance was measured with increasing increments of 0.5 V from 2.5 V, at which polarization switching started. As shown in the R–V hysteresis loop in Figure 4f, resistance switching is observed after 3 V. As the amplitude reaches 3 V, barrier modulation occurs through polarization reversal from the OFF state to the ON state, or vice versa. Finally, resistance switching due to the polarization is complete at ±5 V. Furthermore, in saturation resistance, when an amplitude less than the programmed amplitude is applied, the resistance does not change until the opposite threshold voltage is reached. Therefore, it can be seen that the MFIS FTJ device has excellent memory characteristics that can clearly distinguish the ON/OFF ratio by controlling the polarization reversal with pulse driving.

**Figure 5:**
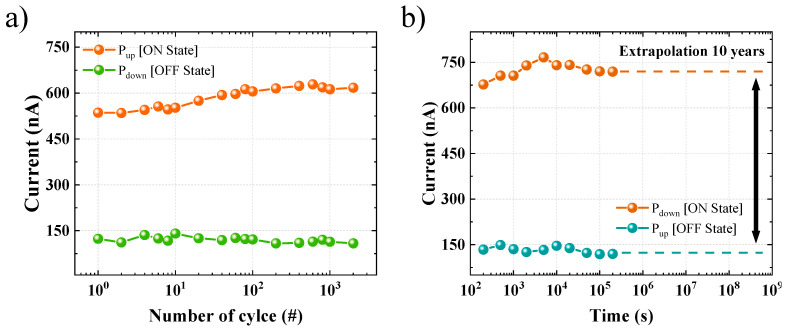
The reliability of the MFIS FTJ device: (**a**) endurance characteristics evaluated by electric field cycling; (**b**) retention characteristics for 10^5^ s.

Endurance and retention tests were performed to evaluate the reliability of the memory characteristics of the previously analyzed FTJ device. Figure 5a shows the endurance properties evaluated by repeated ferroelectric polarization reversal through electric field cycling. The memory characteristics in the pristine state, with a tunneling current difference of approximately four times, are maintained during 2 × 10^3^ electric field cycles. However, the ON/OFF current ratio increases as the current value in the ON state gradually increases during electric field cycling. This can be caused by the formation of leakage current paths due to the rearrangement or creation of defects, such as oxygen vacancies in the ferroelectric film during electrical cycling. In order to secure the reliability of the device, there is a need to continue efforts to minimize the electrical degradation characteristics due to the fatigue phenomenon of the thin film. Nevertheless, the FTJ device still has a wide memory window. Figure 5b shows the retention characteristics to demonstrate the non-volatility of the device. In both the ON and OFF states, the current decay phenomenon in which the non-volatile characteristics disappear is not observed before 10^5^ s, demonstrating that the device has excellent non-volatile characteristics. The extrapolation of this analysis makes it possible to predict that the programmed memory will be maintained for approximately 10 years.

## 4. Conclusions

We demonstrated FTJ devices based on TiN/HSO/TiN (MFM) and TiN/HSO/SiON/Si (MFIS) structures for memory applications. Ferroelectric polarization switching and energy potential barrier modulation were induced by a voltage pulse driving scheme with varying pulse amplitudes or pulse widths. The thin dielectric layer at the interface between the ferroelectric barrier and the metal electrode formed an asymmetric energy barrier in the FTJ device, maximizing the height difference of the barrier during polarization switching; consequently, the TER effect was greatly improved. In addition, this contributed to securing a sufficient memory read driving margin owing to the voltage dividing effect between the ferroelectric film and the insulator and induced stable polarization switching characteristics compared to the symmetrical FTJ device. The MFIS-structured FTJ memory devices can endure over 2 × 10^3^ electric field cycles at the writing program voltage pulse. Moreover, the ON and OFF states of the FTJ device are expected to be maintained for more than 10 years at room temperature, based on the retention data of 10^5^ s. These FTJs with an inter-dielectric layer have a high potential for next-generation non-volatile memory applications.

## Data Availability

The data presented in this study are contained within the article.
